# Simultaneous high‐resolution T_2_‐weighted imaging and quantitative T _2_ mapping at low magnetic field strengths using a multiple TE and multi‐orientation acquisition approach

**DOI:** 10.1002/mrm.29273

**Published:** 2022-05-12

**Authors:** Sean C. L. Deoni, Jonathan O'Muircheartaigh, Emil Ljungberg, Mathew Huentelman, Steven C. R. Williams

**Affiliations:** ^1^ Advanced Baby Imaging Lab Rhode Island Hospital Providence Rhode Island USA; ^2^ Department of Diagnostic Radiology Warren Alpert Medical School at Brown University Providence Rhode Island USA; ^3^ Department of Pediatrics Warren Alpert Medical School at Brown University Providence Rhode Island USA; ^4^ Centre for the Developing Brain School of Biomedical Engineering & Imaging Sciences, Kings College London London UK; ^5^ Department of Perinatal Imaging and Health Kings College London London UK; ^6^ MRC Centre for Neurodevelopmental Disorders Kings College London London UK; ^7^ Department of Medical Radiation Physics Lund University Lund Sweden; ^8^ Department of Neuroimaging Kings College London London UK; ^9^ Neurogenomics Division Translational Genomics Research Institute Phoenix Arizona USA

**Keywords:** child brain development, low field MRI, magnetic resonance imaging, pediatric neuroimaging

## Abstract

**Purpose:**

Low magnetic field systems provide an important opportunity to expand MRI to new and diverse clinical and research study populations. However, a fundamental limitation of low field strength systems is the reduced SNR compared to 1.5 or 3T, necessitating compromises in spatial resolution and imaging time. Most often, images are acquired with anisotropic voxels with low through‐plane resolution, which provide acceptable image quality with reasonable scan times, but can impair visualization of subtle pathology.

**Methods:**

Here, we describe a super‐resolution approach to reconstruct high‐resolution isotropic T_2_‐weighted images from a series of low‐resolution anisotropic images acquired in orthogonal orientations. Furthermore, acquiring each image with an incremented TE allows calculations of quantitative T_2_ images without time penalty.

**Results:**

Our approach is demonstrated via phantom and in vivo human brain imaging, with simultaneous 1.5 × 1.5 × 1.5 mm^3^ T_2_‐weighted and quantitative T_2_ maps acquired using a clinically feasible approach that combines three acquisition that require approximately 4‐min each to collect. Calculated T_2_ values agree with reference multiple TE measures with intraclass correlation values of 0.96 and 0.85 in phantom and in vivo measures, respectively, in line with previously reported brain T_2_ values at 150 mT, 1.5T, and 3T.

**Conclusion:**

Our multi‐orientation and multi‐TE approach is a time‐efficient method for high‐resolution T_2_‐weighted images for anatomical visualization with simultaneous quantitative T_2_ imaging for increased sensitivity to tissue microstructure and chemical composition.

## 1 INTRODUCTION

Quantitative measurement of the transverse magnetic relaxation time constant (T_2_) is a valuable tool in both clinical and research MRI imaging applications. Clinically, these uses include visualizing spinal disc degeneration,^1^ diagnosis of cardiomyopathy,^2^ liver iron quantification,^3^ and evaluation of articular cartilage health and degeneration.^4^ In the research context, quantitative (q)T_2_ imaging has been used to investigate white matter maturation and myelination during neurodevelopment,^5^ white and gray matter microstructure degeneration associated with aging and cognitive decline (including Alzheimer disease),^6^ brain iron changes associated with neurological disorders (e.g., Parkinson disease),^7^ and quantifying hypoxic–ischemic injury in neonates^8^ among other uses.

Conventionally, qT_2_ imaging is performed with a multi‐echo fast or turbo spin‐echo (FSE or TSE) approach, with T_2_ calculated at each voxel or region‐of‐interest from two or more images acquired with different *effective* echo times, TE,^9^ the time at which the central k‐space lines are acquired. Under ideal conditions, i.e., single‐species relaxation and full suppression of stimulated or indirect echoes, T_2_ can be calculated from two acquired images at different TEs using a linearized fit to the signal equation

(1) $$S\left(\mathrm{TE}\right)=M_{0}e^{-\frac{\mathrm{TE}}{T_{2}}}.$$

Past work has eloquently shown that many tissues, including brain white matter, are more accurately modeled as the summation of multiple T_2_ species that reflect distinct tissue components (i.e., myelin vs the intra‐ and extra‐cellular water in brain white matter).^10^, ^11^ In these tissues, the calculated two‐point T_2_ will depend on the chosen TEs and will be biased toward the faster‐relaxing myelin‐associated water or the slower‐relaxing intra‐ and extra‐cellular water.^12^ However, even under these conditions, a single‐component qT_2_ measurement can still provide useful information on tissue change and damage with high intra‐ and inter‐site reproducibility provided matched acquisition parameters.^13^, ^14^

Widespread adoption of quantitative imaging has been limited in part by the lengthy acquisition times needed to reliably calculate high‐resolution qT_2_ maps. This limitation is magnified at lower field strengths (e.g., 50–200 mT) where the SNR of the acquired T_2_‐weighted images is further reduced. Here, images are often acquired with non‐isotropic voxels with large through‐plane dimensions (e.g., 1.5 mm × 1.5 mm × 5 mm) to reduce imaging time while maintaining acceptable SNR. Lower field strength systems, such as the Hyperfine Swoop (64 mT) and others, offer the potential for a new approach to clinical and research imaging in which the scanner is brought to the patient or participant.^15^ Portable neuroimaging could be transformative for clinical studies of neonatal hypoxic–ischemic encephalopathy, in which unstable infants are difficult to transport between the neonatal intensive care and radiology units; or population‐based studies of Alzheimer disease, in which many interested individuals are unable to participate due to challenges with mobility and transportation to an imaging center.

Currently, available product sequences on the Hyperfine system are set to a default spatial resolution of (1.5 mm × 1.5 mm × 5 mm). A research agreement with the manufacturer has allowed us to adjust this base resolution but, unfortunately, higher resolution isotropic images yield lengthy acquisition times (e.g., ∼12–15 min for a single 2 mm × 2 mm × 2 mm T_2_‐weighted image). Thus, acquisition of even just two differing TEs may be prohibitively long in many clinical settings or in sensitive (infant or elderly populations). The inability to remain motionless for this entire scan and exam will result in likely motion‐related artifact corruption in non‐sedated infants and all but the most exceptionally compliant clinical populations.

One approach that has found considerable success in addressing the challenge of time‐efficient high‐resolution imaging is super‐resolution (SR) reconstruction from multiple lower resolution anisotropic images acquired in three or more rotated orientations (i.e., axial, sagittal, and coronal).^16^, ^17^ While this does not necessarily reduce total imaging time, each individual acquisition is shorter and potentially more tolerable in motion‐sensitive populations (and each can be repeated independently without requiring the full exam to be restarted).

In conventional SR, each image is acquired with similar acquisition parameters except for orientation. However, varying the TE of each acquisition may further allow qT_2_ calculation without loss of image quality or increase in imaging time. Such an approach has been previously demonstrated for qT_1_ imaging.^18^

The aim of this work, therefore, was to investigate the feasibility of this simultaneous multi‐TE and multi‐orientation approach for the concurrent collection of high‐resolution isotropic T_2_‐weighted and qT_2_ imaging at low field (64 mT) on a Hyperfine Swoop system. We show that reliable isotropic qT_2_ imaging is possible with a clinically tolerable 12‐min scan time and with quantitative values that agree well with gold‐standard reference measures. While further work is needed to improve overall image quality, these results represent an important advance for low field neuroimaging.

## 2 METHODS

### 2.1 Approach

Super‐resolution and related algorithms aim to improve the spatial resolution of an image by combining information from one or more lower‐resolution images. While the initial applications of SR to MRI dates back almost two decades,^19^ its use has accelerated over the past 3–5 y with the development of new deep learning techniques (e.g., Refs. [20, 21, 22]) In many of the original MRI implementations, the lower resolution images were acquired with subtle shifts in one or more directions.^23^, ^24^, ^25^ In more recent implementations, often associated with fetal MRI,^16^, ^26^, ^27^ the lower resolution images are acquired from different orientations—often chosen as the three principal axes (axial, sagittal, and coronal). To reconstruct a high‐resolution image, the general approach follows an initial registration of the low‐resolution images to each other in a common registration space to create a template image with the desired spatial resolution with a maximum likelihood approach used to interpolate the high‐resolution image intensities.^24^, ^28^ Iterative affine and non‐linear transformations are then performed between the source data and the template, with the template improved at each step.

**FIGURE 1:**
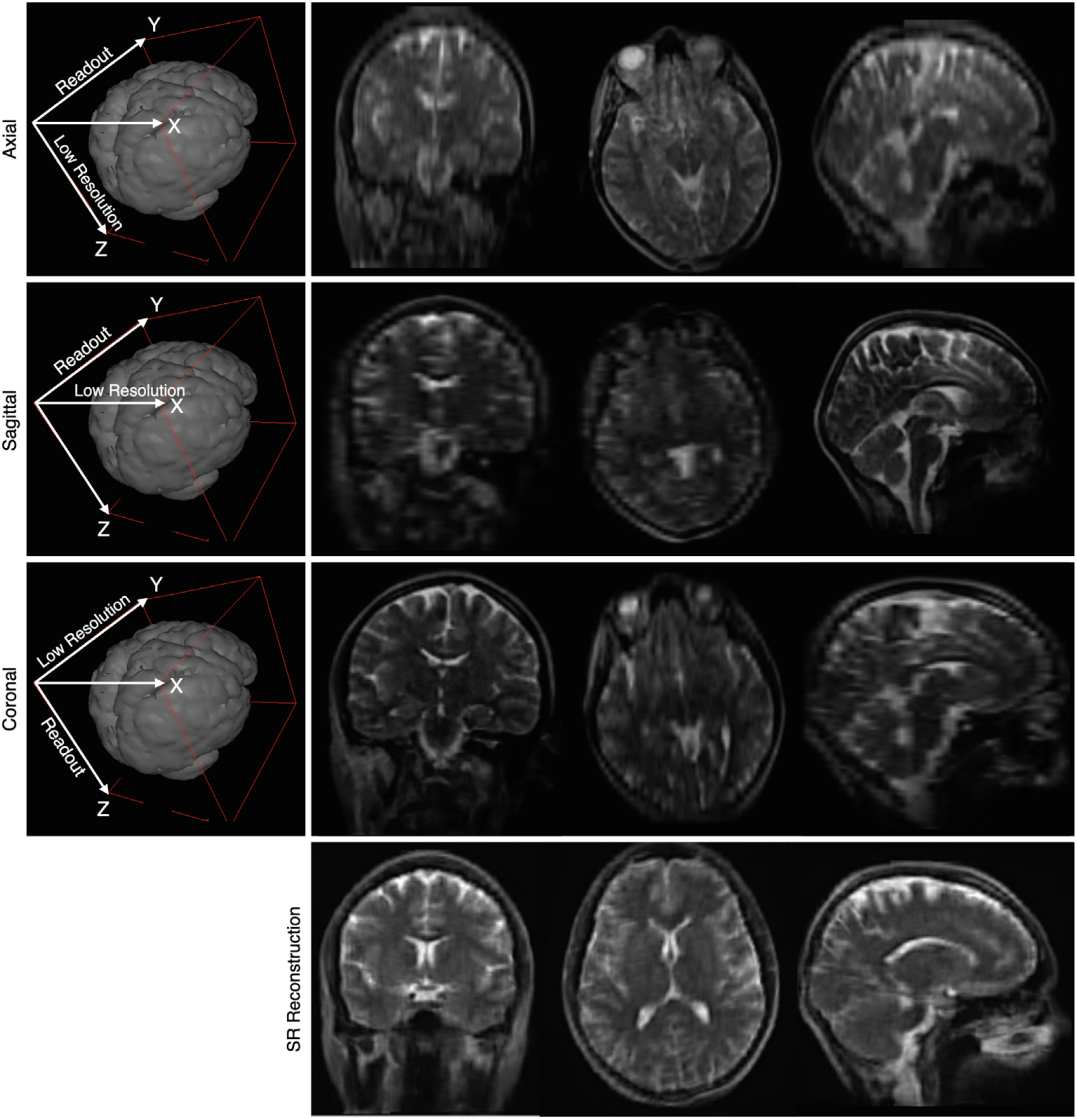
Example illustration of SR image reconstruction using images acquired in the axial, sagittal, and coronal orientations (the frequency encoding/readout and lower resolution directions are labeled) and the final reconstructed image

A simplified approximation to SR can be achieved through repeated multi‐resolution registration as implemented in the Advanced Normalization Tools (ANTS) multivariate template construction tool^29^ as described in Ref. [30]. Here, the low‐resolution images are aligned using linear and diffeomorphic registration with symmetric normalization. This approach provides not only the combined high‐resolution isotropic image but also the forward and inverse transformations for each low‐resolution image to the combined result (Figure 1).

For our proposed approach, anisotropic T_2_‐weighted axial, sagittal, and coronal images are acquired with incremented TEs. In‐plane resolution for each image is 1.5 mm × 1.5 mm with a through‐plane resolution of 5 mm (like shown in Figure 1). Using the approach described, these data are first combined into a single (1.5 × 1.5 × 1.5) mm^3^ T_2_‐weighted image for anatomical visualization. A conventional non‐linear exponential fit for T_2_ is then performed using the three registered images in the high‐resolution space to generate the qT_2_ image.

### 2.2 Data collection

To validate our approach, a series of phantom and in vivo human imaging data were acquired on a 64 mT Hyperfine Swoop system. All human data were acquired following informed consent as part of an approved study monitored by our institution's ethical review board. Phantom measurements were acquired of the commercially available CaliberMRI phantom that is closely modeled on the NIST “Phannie” phantom^31^ and provides a range of T_1_ and T_2_ values. In vivo data were acquired of five healthy female adult volunteers with a mean age of 24 ± 3 y.

Three datasets were acquired of the phantom and each volunteer (described in Table 1):

1. “Gold Standard” *Hyperfine multi‐TE* T_2_: single‐orientation FSE approach with five incremented TEs.
2. *Single orientation multi‐TE* FSE approach with data collected at three incremented TEs of approximately 123, 182, and 242 ms (rounded to the nearest ms).
  and
3. Our proposed multi‐orientation and multi‐TE approach with FSE images acquired with the same three TEs as^2^ but acquired in the axial, coronal, and sagittal orientations, respectively.

**TABLE 1:** Acquisition parameters for the three sets of data collected phantom and each human volunteer

Hyperfine multi‐TE T2	TE #1	TE #2	TE #3	TE #4	TE #5
Axial FOV (X × Y × Z)cm^3^	16.8 × 20.4 × 18.0	16.8 × 20.4 × 18.0	16.8 × 20.4 × 18.0	16.8 × 20.4 × 18.0	16.8 × 20.4 × 18.0
Matrix (X × Y × Z)	112 × 136 × 36	112 × 136 × 36	112 × 136 × 36	112 × 136 × 36	112 × 136 × 36
Readout direction	Y (AP)	Y (AP)	Y (AP)	Y (AP)	Y (AP)
In‐plane resolution (mm × mm)	1.5 × 1.5	1.5 × 1.5	1.5 × 1.5	1.5 × 1.5	1.5 × 1.5
Slice thickness (mm)	5	5	5	5	5
TR (ms)	2200	2200	2200	2200	2200
TE (ms)	60	100	150	200	300
ETL	12	20	30	40	60
Acquisition time (min)	13:37				
Single‐orientation multi‐TE	TE #1	TE #2	TE #3		
Axial FOV (X × Y × Z) cm^3^	18.0 × 21.9 × 18.0	18.0 × 21.9 × 18.0	18.0 × 21.9 × 18.0		
Matrix (X × Y × Z)	120 × 146 × 36	120 × 146 × 36	120 × 146 × 36		
Readout direction	Y (AP)	Y (AP)	Y (AP)		
In‐plane resolution (mm × mm)	1.5 × 1.5	1.5 × 1.5	1.5 × 1.5		
Slice thickness (mm)	5	5	5		
TR (ms)	2000	2000	2000		
TE (ms)	122.8	182.4	241.6		
ETL	20	30	40		
Acquisition time (min)	5:58	4:00	3:00	12:58	
Multiple‐orientation multi‐TE	TE #1	TE #2	TE #3		
Axial FOV (X × Y × Z) cm^3^		18.0 × 21.9 × 18.0			
Sagittal FOV (X × Y × Z) cm^3^	18.0 × 21.9 × 18.0				
Coronal FOV (X × Y × Z) cm^3^			18.0 × 18.0 × 20		
Matrix (X × Y × Z)	120 × 146 × 36	120 × 146 × 36	120 × 120 × 40		
Readout direction	Y (AP)	Y (AP)	Z (SI)		
In‐plane resolution (mm × mm)	1.5 × 1.5	1.5 × 1.5	1.5 × 1.5		
Slice thickness (mm)	5	5	5		
TR (ms)	2000	2000	2000		
TE (ms)	122.8	182.4	241.6		
ETL	20	30	40		
Acquisition time (min)	4:11	4:00	3:30	11:41	

In all cases, the T_2_ data were collected using a fully 3D sequence with a Cartesian k‐space trajectory. The built in image reconstruction pipeline was used that includes gradient non‐linearity correction and noise compensation using external magnetic field monitors. For the Hyperfine multi‐TE approach (#1), the data are acquired sequentially with the qT_2_ map calculated using an exponential fit. For approaches #2 and #3 in which each TE image was acquired and reconstructed independently and then the qT_2_ map calculated, the deep learning reconstruction approach (similar to that described in Ref. [32]) was turned off in favor of a conjugate gradient least square method (e.g., Ref. [33]) to avoid unknown scaling and manipulation of the signal values between the different TE acquisitions. To vary the TEs, the echo train length (ETL) was changed from 20 for the shortest TE, 30 for mid‐length TE, and 40 for the longest TE. Given the low field strength and low risk of high energy deposition (specific absorption rate, SAR), the excitation and refocusing pulses were 90 and 180 degrees, respectively.

Image orientations for the short, mid, and long TE acquisitions were chosen to minimize overall acquisition time, yielding a total scan time of just under 12 min.

### 2.3 Data analysis and comparison

For the Hyperfine multi‐TE approach, the qT_2_ maps were automatically calculated using on‐scanner software that consisted of an initial linearized least squares estimation to provide an initial estimate of the model parameters, and then a single exponential fit to the data (assuming Equation (1)).

For the single orientation multi‐TE approach, linear image alignment was first performed to account for any subject movement between images followed by T_2_ calculations at each imaging voxel using a similar approach of an initial linearized least squares estimation with a non‐linear fit to the exponential model using a Nelder–Mead Simplex method with 100 sequential iterations. For the multiple orientation multi‐TE approach, SR reconstruction was first used to calculate the isotropic spatial resolution images, and then T_2_ values were estimated using linearized least squares and non‐linear exponential fit using a Nelder–Mead Simplex. Example analysis code for these steps is provided as supporting Information Appendix S1.

Given the low SNR of the acquired images, we sought to evaluate the use of anisotropic noise filtering^34^ to improve map quality. For the multiple‐orientation multi‐TE approach we applied the spatially adaptive denoising approach of Manjon et al.^35^ to the aligned high spatial resolution multi‐TE source images after SR reconstruction but prior to T_2_ calculation and then followed the same steps as above to calculate the qT_2_ image.

Following calculation of the qT_2_ images from each of the three datasets, mean measures were obtained from 10 phantom elements and six in vivo brain regions: right and left anterior internal capsule white matter, right and left posterior white matter, cerebellar white matter, and body of the corpus callosum. In vivo region masks were first manually drawn on the MNI template, which was then non‐linearly aligned to each participant's T_2_‐weighted image and superimposed onto their corresponding qT_2_ image. Agreement between the measures obtained with the different approaches was then assessed via the intra‐class correlation, ICC.

## 3 RESULTS

**FIGURE 2:**
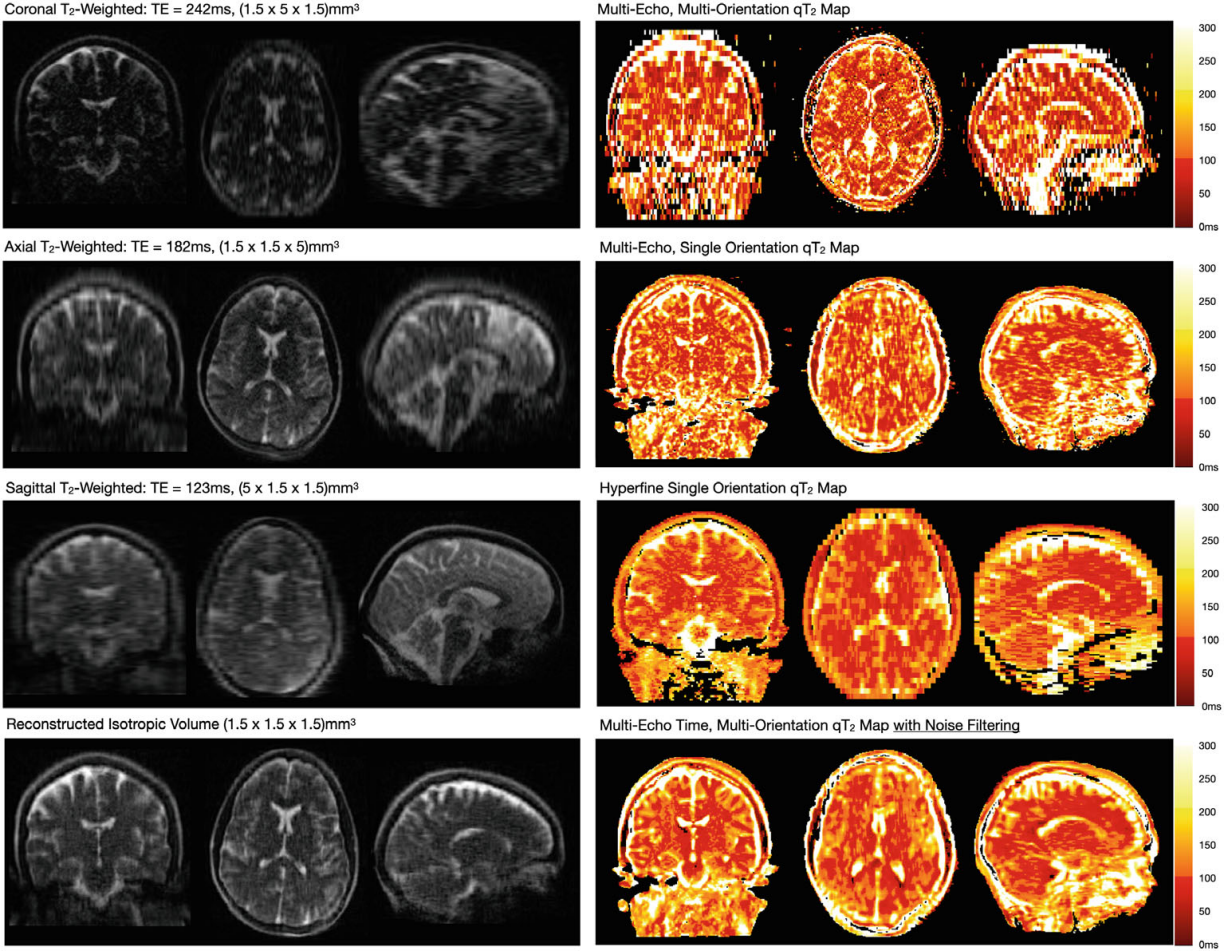
Analysis workflow from the acquisition of the source anisotropic T_2_‐weighted data with coronal, axial, and sagittal orientations (left panel); SR reconstruction of an isotropic T_2_‐weighted image from the acquired data (bottom, left panel); and calculation of qT_2_ maps from the aligned and resampled multi‐orientation data (top, right panel). For comparison, qT2 maps calculated from multiple TEs acquired in a single orientation and using Hyperfine‐provided five‐TE approach (middle, right panel) are also shown. As well, we show a multi‐orientation + multi‐TI qT2 calculated from data that was preprocessed using adaptive denoising (bottom, right panel)

**FIGURE 3:**
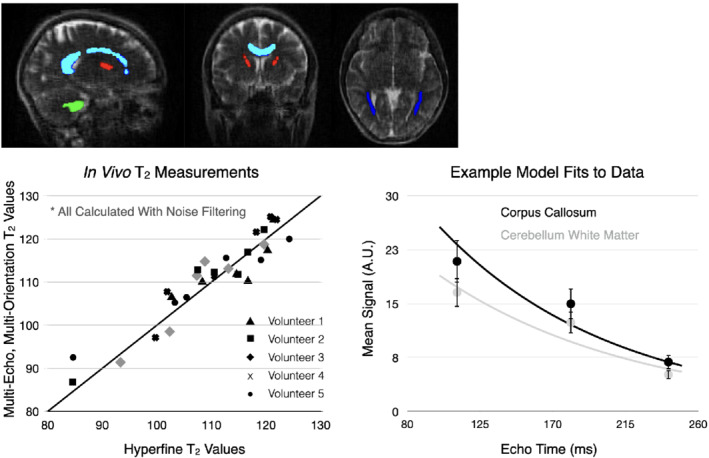
Comparison of qT_2_ values from four brain regions of interest (top) in each healthy volunteer. Brain regions included cerebellar white matter (green), corpus callosum (light blue), anterior internal capsule (red), and posterior thalamic radiations (dark blue). The ICC between the noise filtered multi‐orientation + multi‐TE and reference Hyperfine 5‐TE qT_2_ values was 0.93. Example monoexponential fits to the corpus callosum and cerebellar white matter data from one of the healthy volunteers is also shown

Figure 2 provides an overview of collected data, processing workflow, and calculated qT_2_ images using each of the acquisition approaches for one of the healthy volunteers. No significant B_0_‐field related distortion effects are noted between the multi‐orientation images, except in the posterior of the sagittal image, which appears to be handled during the non‐linear reconstruction process. A visual comparison of in vivo T_2_ values derived from the noise‐filtered multi‐orientation + multi‐TE data are provided in Figure 3, with the values calculated using the Hyperfine five‐TE approach assumed as reference values. Overall, we note strong alignment between the multi‐orientation + multi‐TE and reference T_2_ values, with an ICC of 0.93. For comparison, the ICC between the non‐noise filtered multi‐orientation + multi‐TE and reference T_2_ values is 0.85.

Examining the Hyperfine and non‐noise filtered images (Figure 2) we note the multi‐orientation + multi‐TE images suffer from significantly reduced SNR, likely owing to the reduced acquisition time (13:37 vs 11:41) and smaller voxel volume (11.25 mm^3^ vs 3.38 mm^3^) of the multi‐orientation + multi‐TE data. Based on conventional associations, these differences should translate to an SNR reduction of approx. 400%, which agrees well with the measured difference in white matter T_2_‐to‐noise ratio of 9.4 in the reference map and 3.2 in the multi‐orientation + multi‐TE map. However, making use of spatially adaptive denoising substantially improves the visual appearance of the qT_2_ maps, despite subtle loss of image detail.

**FIGURE 4:**
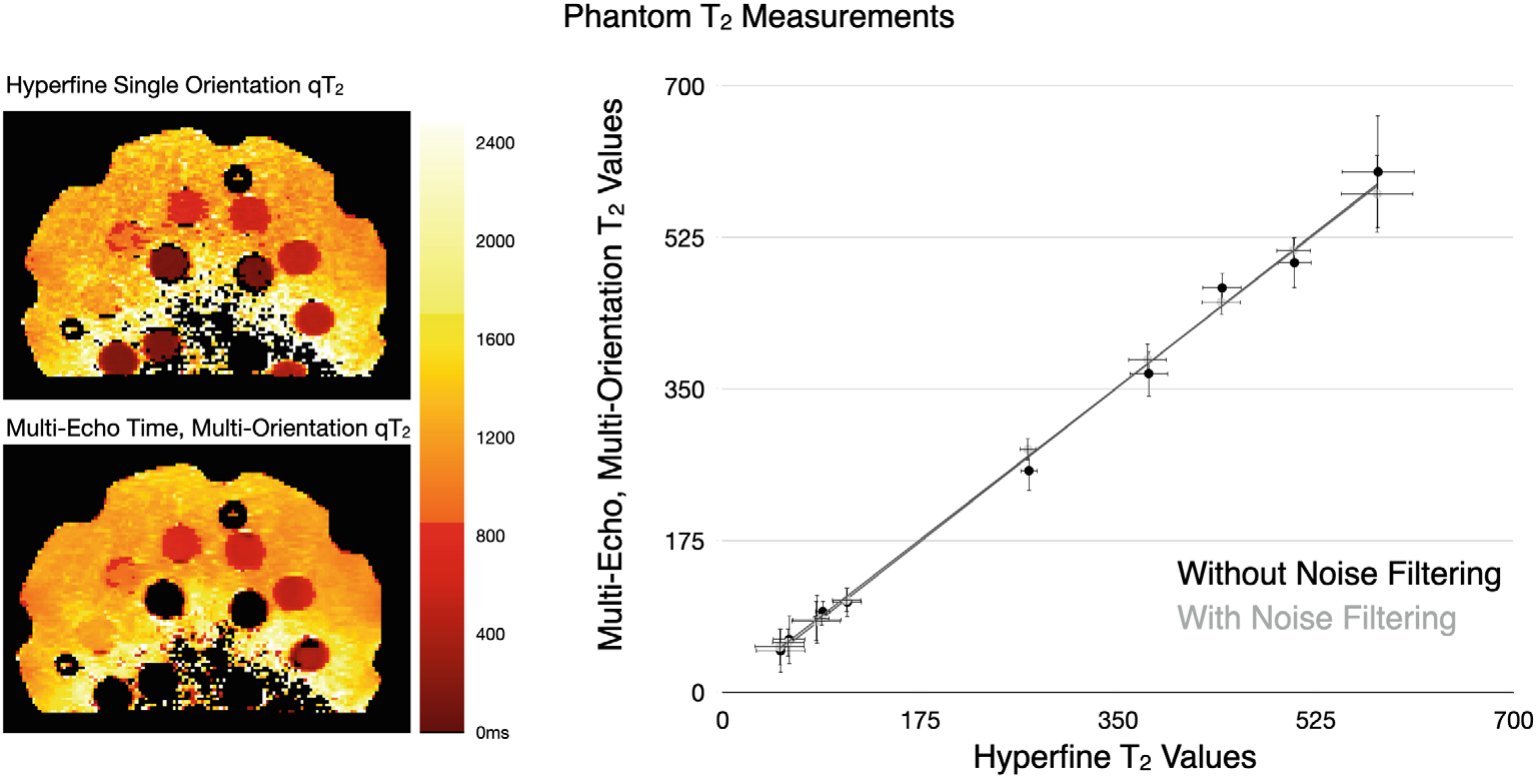
(Left) Comparison of qT_2_ images through the multi‐element phantom and (Right) Mean phantom multi‐orientation + multi‐TE and reference Hyperfine 5‐TE qT_2_ values. The solid line corresponds to the line of unity, and errors bars in the phantom measurements denote 1 SD. ICC values for the phantom data were 0.97 (with denoising) and 0.96 (without denoising)

Building on the in vivo results in Figures 2 and 3, Figure 4 displays a comparison of the Hyperfine five‐TE and noise‐filtered multi‐orientation + multi‐TE qT_2_ maps and mean phantom element T_2_ values. As with the in vivo data, we find agreement between the values, with an ICC of 0.96.

From the in vivo data, mean T_2_ values for the different regions of interest were: cerebellar white matter, 110 ± 19 ms; corpus callosum, 112 ± 7 ms; left frontal white matter, 114 ± 3.5 ms; right frontal white matter, 118 ± 6 ms; left posterior white matter, 98 ± 7 ms; and right posterior white matter, 102 ± 9 ms. These measures are in general agreement with those previously reported at 1.5 and 3 T (ranging from 60 to 130 ms),^36^ as well as with prior measurements at lower field strengths (80–130 ms at 50–150 mT).^37^, ^38^ The relative invariance of in vivo T_2_ measures with field strength is theoretically predicted.^39^

## 4 DISCUSSION

In past work, our group investigated the use of portable low field MRI for pediatric neuroimaging,^40^ demonstrating the ability to reliably image and quantify total and regional brain volumes. That analysis, however, used the conventional raw anisotropic T_2_‐weighted images provided by the Hyperfine system, and volumes were over‐estimated relative to higher field strength isotropic images. The approach proposed here offers important advantages over this earlier work. Specifically, the reconstruction of isotropic resolution images may help address this estimation bias and improve overall comparisons with high field strength data. Recently, a joint SR and image synthesis approach has been proposed to allow reconstruction of high‐resolution 1 mm isotropic images from anisotropic and low‐resolution data, such as that acquired with Hyperfine.^41^ We speculate that improved image synthesis will be possible from the higher resolution low field images provided through our approach. Of note, the open‐source SynthMR package makes use of low field T_1_ and T_2_ images to reconstruct the synthetic high‐resolution T_1_ weighted image using the predict_command_line_hyperfine.py module. The acquisition time for these is approximately 11‐min—approximately the same as for our approach.

One of the principal limitations of our approach is the relatively low SNR of the calculated T_2_ map. For clinical studies, this may translate to a need for larger samples sizes to identify subtle differences in neurodevelopment, accelerated degeneration, increased iron accumulation, or other examples. Improvements to the qT_2_ map quality may be possible by down‐sampling the raw data used for the qT_2_ map calculation, although at the obvious cost of resolution. Our use of noise filtering and reduction, however, also provides a significant improvement in image quality without degrading T_2_ measurement accuracy. Additional alternatives, image synthesis and deep‐learning may also offer methods to not only improve the single‐component qT_2_ images presented here but also model multi‐component relaxometry and myelin water imaging.^42^

A second limitation of our approach is the relatively long TEs used relative to the TRs of the tissue we are measuring. Ideally, we would aim to sample the decay curve more completely, including shorter TEs.^43^ Unfortunately, we are relatively limited in terms of available sequence modifications on the Hyperfine system and balancing the range of TEs with acquisition time yielded the values used here (approximately 123, 182, and 242 ms). While the mean fits to the data (examples shown in Figure 3) appear valid, it is hoped that greater access to pulse sequence design will allow greater flexibility in chosen TEs, further improving the qT_2_ accuracy and image quality.

Greater sequence design access may also allow for additional subtle control of FOV positioning, which is currently performed automatically. By varying not only orientation, as done here, but also adding sub‐voxel shifts along the other image dimensions may further improve the SR reconstruction, allowing further reduction of voxel dimensions below the (1.5 × 1.5 × 1.5) mm^3^ achieved here.^19^

While our presented results are preliminary and are demonstrated in adult subjects only, they illustrate the potential of low field imaging as a viable complement to high field systems. Furthermore, they offer the potential for imaging studies of neurodevelopment or neurodegeneration in many low‐ and middle‐income settings where disease, malnutrition, psychosocial adversities, and other environmental exposures may profoundly affect brain structure and function, but where access to MRI is significantly limited.

## 5 CONCLUSIONS

It is increasingly clear that portable low field imaging offers a new paradigm in neuroimaging. Not only allowing increased access to clinical and research populations in the “global north,” but affording the opportunity for accessible imaging in low‐ and middle‐income settings. At present, however, commercial low field strength devices are limited in the range of available image contrasts and acquisition methods. Here, we have taken an initial step toward acquiring higher resolution T_2_‐weighted imaging for anatomical visualization with simultaneous qT_2_ imaging for increased sensitivity to tissue microstructure and chemical composition, all performed within a clinically manageable 12 min. It is envisaged that, as low field devices become more commonplace, and imaging data are shared across the research community, improvements to the described technique will allow for increased performance, lower noise, and more rapid acquisition—further enhancing the diagnostic capability of these devices.

## CONFLICT OF INTEREST

The authors report no significant financial conflicts of interest with respect to the subject matter of this manuscript.

## Supporting information


Appendix S1.
